# Evaluating catastrophic health expenditures among patients with long bone fractures in Ghana's major teaching hospitals: a hospital-based analysis

**DOI:** 10.1186/s12913-025-12250-6

**Published:** 2025-01-15

**Authors:** Alexis D. B. Buunaaim, Michel Adurayi Amenah, Dominic Konadu-Yeboah, Richard Baidoo, Amgbo Asare, Wilfred Larbi Addo, Claude Martin

**Affiliations:** 1 School of Medicine, University for Development Studies, Tamale, Ghana; 2 Department of Surgery-Orthopaedics Unit, Tamale Teaching Hospital, Tamale, Ghana; 3 Public Health Department, NIHR Global Health Research Centre for Non-Communicable Disease Control in West Africa, Ghana College of Physicians and Surgeons, Accra, Ghana; 4 Division of Health Research, Lancaster University, Lancaster, UK; 5 School of Medicine and Dentistry, Kwame Nkrumah University of Science and Technology, Kumasi, Ghana; 6 Department of Surgery, Komfo Anokye Teaching Hospital, Kumasi, Ghana; 7 School of Medical Sciences, University of Cape Coast, Cape Coast, Ghana; 8 Department of Surgery, Cape Coast Teaching Hospital, Cape Coast, Ghana; 9 Department of Trauma and Orthopaedics, Korle-Bu Teaching Hospital, Accra, Ghana; 10 St. Joseph’s Hospital, Koforidua, Ghana; 11 AO Alliance, Stiftung, Switzerland

**Keywords:** Catastrophic health expenditures, Long bone fractures, Out-of-pocket payments

## Abstract

**Background:**

In low and middle-income countries like Ghana, out-of-pocket (OOP) payments remain a significant barrier to healthcare access, often leading to catastrophic health expenditures (CHE). This study evaluates the incidence of CHE among patients treated for long bone fractures at Ghana’s major teaching hospitals, providing insight into the economic burdens faced by these patients.

**Methods:**

This cross-sectional study analyzed data from 2,980 patients with long bone fractures treated at four major teaching hospitals in Ghana from July 2017 to July 2020. We collected demographic, clinical, and economic data, including OOP payments and patient-reported income, to assess the incidence of CHE at varying income thresholds (10%, 20%, 30%, 40%). Logistic regression models were used to identify predictors of CHE, with variables including age, gender, education, region, fracture type, injury severity, and NHIS coverage.

**Results:**

The incidence of CHE was highest at the 10% income threshold (53.21%) and decreased with higher thresholds. Male patients incurred higher average OOP payments ($343.68) than females ($271.63), and patients with tibia fractures faced the highest financial burden. Educational and regional disparities were evident, with lower CHE rates among patients with higher educational attainment and those from northern regions. NHIS coverage provided limited financial relief, particularly at lower income thresholds.

**Conclusion:**

Long bone fractures impose a substantial financial burden on patients in Ghana, with significant gender, educational, and regional disparities in OOP payments and CHE. While NHIS provides some relief, it remains inadequate in protecting patients from financial distress. Policy interventions aimed at expanding NHIS coverage, reducing OOP payments for high-cost treatments, and addressing geographic inequities are urgently needed to improve financial protection for patients with long bone fractures in Ghana. Future research should focus on capturing long-term financial impacts and improving income data accuracy to better inform healthcare policies.

## Introduction

Healthcare financing remains a critical challenge in low and middle-income countries (LMICs), where out-of-pocket (OOP) payments constitute a significant portion of health expenditure [1]. In Ghana, despite efforts to improve health financing mechanisms through National Health Insurance Schemes, many patients still face high OOP payments, particularly for specialized treatments in teaching hospitals [2, 3]. Catastrophic health expenditure, defined as health spending that threatens a household's ability to maintain its subsistence needs, is a key indicator of financial protection in health systems [4, 5]. Typically, expenditures exceeding 10% or 20% of household yearly gross income are considered catastrophic, depending on the specific thresholds set by different studies. This threshold-based approach helps identify the extent to which medical expenses can deplete household resources, potentially leading to impoverishment or forcing families to forego other basic needs [1, 2].

The relevance of studying CHE in the context of long bone fractures is underscored by the nature of these medical emergencies requiring immediate or urgent care [6]. Long bone fractures often require immediate, high-intensity and skilled medical intervention, including surgery, hospitalization, and long-term rehabilitation, which can lead to substantial healthcare costs. In Ghana, where many rely on manual labor to earn a living, the incapacitation caused by a fracture can double the financial strain by not only increasing medical costs but also reducing income due to work absences [1, 7].

Several studies have documented the prevalence and impact of CHE across various contexts and medical conditions, providing valuable insights into the financial burdens faced by patients and their families. One study found that 47.3% of hospitalized patients in Iran faced CHE, with hospital type and insurance status being significant factors influencing these expenditures [8]. Similarly, another study reported that 22.2% of patients in India experienced CHE following injury hospitalization, and 12.2% of these patients fell below the poverty line due to the financial burden of their medical expenses [9]. Similarly, Egyptian households experienced an OOP health payment, exacerbating the poverty gap by 1.4% [10]. Further emphasizing the widespread impact of CHE, another study noted that 33.1% of elderly patients in Lucknow, India, incurred CHE when seeking in-patient care [11].

Orthopaedic conditions, particularly long bone fractures, are associated with significant medical costs, often leading to CHE. Public insurance in the United States does not adequately cover hospital costs for long bone fractures, causing patients with public insurance to travel further for care compared to those with commercial insurance [12]. Another study reported that 91.5% of uninsured patients and 10.1% of privately insured patients faced CHE after motor vehicle crashes leading to fractures [13].

In Ghana, some studies have examined CHE, highlighting the significant financial burden faced by households due to OOP healthcare payments. The Ghana National Health Insurance Scheme (NHIS), introduced in 2003, aimed to mitigate these financial barriers, but studies reveal persistent challenges. For instance, insured patients still incur substantial OOP costs, with a high incidence of CHE observed among the poor and vulnerable populations [14, 15]. The NHIS's coverage gaps and the limited response to patient reports further exacerbate these issues [16, 17]. Research has shown that even with insurance, a significant proportion of households face CHE due to various medical expenses, including maternal healthcare and surgical costs [18–20]. While these studies provide crucial insights into the financial strain of healthcare on Ghanaian households, there is a noticeable gap in the literature specifically addressing the economic impact of long bone fractures.

This study aims to fill this gap by focusing on a unique and understudied area, the financial burden of long bone fractures in Ghana. It provides new insights into the incidence and determinants of CHE among patients with long bone fractures, contributing to the broader understanding of how high-cost, specialized treatments impact household finances. Moreover, this research has direct policy relevance, as it highlights areas where the existing National Health Insurance Scheme (NHIS) coverage is insufficient to protect patients from financial distress. By identifying specific factors contributing to CHE in this patient group, the study lays the groundwork for policy recommendations aimed at reducing OOP payments for orthopaedic care and improving financial protection under the NHIS.

This study seeks to fill this gap by evaluating the extent and determinants of CHE among patients with long bone fractures treated at major teaching hospitals in Ghana, thereby contributing to a more nuanced understanding of the financial challenges associated with orthopaedic care in the country. Moreover, by identifying the specific economic impacts of treating long bone fractures in teaching hospitals, this research could inform targeted policy interventions. These could include improvements in health insurance coverage, subsidies for high-cost procedures, or the development of programs aimed at reducing OOP expenditures for vulnerable populations. The rest of the paper is organized as follows: "Methods" section presents the methodology used in the study, including data collection, sample selection, and analysis. "Results" section details the results, focusing on the incidence and determinants of CHE. "Discussion" section discusses the findings in relation to existing literature and provides policy recommendations. Finally, "Conclusion" section concludes the study with reflections on the implications for health financing and potential areas for future research.

## Methods

### Study setting

The study was conducted at four major teaching hospitals in Ghana, strategically selected to offer a comprehensive overview of trauma care services. These hospitals include Cape Coast Teaching Hospital (CCTH) in the Central Region, serving as a primary referral center; Komfo Anokye Teaching Hospital (KATH) in Kumasi, the main referral center for the Ashanti Region; Korle Bu Teaching Hospital (KBTH) in Accra, the largest in Ghana, catering to the Greater Accra Region and beyond; and Tamale Teaching Hospital (TTH) in the Northern Region, focused on providing essential trauma care to a diverse population [21].

### Inclusion criteria

The study included individuals of all ages who reported to sentinel sites with long bone fractures, specifically of the humerus, radius, ulna, femur, or tibia. This inclusive approach ensured the comprehensive capture of trauma cases across diverse age groups and demographics [21].

### Eligibility procedure

Trained research officers identified eligible patients in emergency rooms, triage, outpatient departments, and other hospital areas, conducting daily scouting to ensure no cases were missed, followed by obtaining informed consent [21].

### Informed consent

Research officers (ROs) orally obtained informed consent, explaining the surveillance procedures to participants or caregivers. For minors, the process was explained to caregivers, with assent sought from the child when applicable [21].

### Data collection and follow-up

All demographic, epidemiological, clinical, and economic burden data were collected using the RedCap database. The research officers, under the supervision of a trauma specialist, recorded information directly from participants and abstracted additional data from patient charts, including fracture classification, evaluations, and imaging reports. Data on economic burden, patient satisfaction, and post-traumatic stress disorder were collected at least one-month post-injury to capture the full extent of healthcare-related costs [21].

### Data source

The study utilized data from a three-year Trauma Registry (July 2017—July 2020) housed within the RedCap database. This registry, strategically established at the four major Ghanaian teaching hospitals, contained all the demographic and economic burden data required for the analysis of catastrophic health expenditures [21]. The data collected was analyzed using STATA 18 [22]. The Trauma Registry has been active since 2017 and enrolled a total of 10,214 patients by the end of 2020. Specifically, 1,428 patients were registered in 2017, 4,586 in 2018, 3,136 in 2019, and 1,064 in 2020. The registry tracks a wide range of trauma cases, including long bone fractures, with an average of approximately 2,500 long bone fractures recorded annually. For this study, we focused on patients with long bone fractures who responded to both income and out-of-pocket payment questions. Out of the total patient cohort, 2,980 patients met these criteria and formed the final sample used in our analysis of catastrophic health expenditures. The data collected included demographic details, clinical characteristics, and economic burden information essential for assessing the incidence and determinants of CHE among these patients.

### Ethical clearance

Ethical clearance to conduct the study was obtained from the Committee on Human Research Publications and Ethics (CHRPE), of Kwame Nkrumah University of Science and technology (Approval number: CHRPE/AP/290/19). The study was conducted in full compliance with the ethical principles outlined in the Declaration of Helsinki and adhered to all applicable national guidelines. Informed consent was obtained from all participants prior to their inclusion in the study.

### Measuring catastrophic healthcare payments

For this study, we utilized the total annual income reported by patients (data was captured for all respondents to this question) and the total OOP expenses incurred for long bone fracture treatment during their hospital stay. All OPP payments made by patients were originally recorded in Ghanaian cedis. To ensure consistency in financial comparisons, these amounts were converted to U.S. dollars using the average exchange rate of GHS 5.08 per dollar for the period from July 2017 to July 2020, sourced from the World Bank database [23]. This conversion ensures that any fluctuations in exchange rates over the study period are accounted for, providing an accurate representation of the financial burden across all patients. This calculation excluded any hospital or treatment costs covered by health insurance and the cost of implants. Using this information, we defined specific indices to measure and evaluate the catastrophic impact of these healthcare expenditures [24, 25].

### Catastrophic payment headcount index

The catastrophic payment headcount index $H$ represents the percentage of patients in the sample whose healthcare expenditures as a proportion of their income exceed a specified threshold ${Z}_{cat}$. Let ${P}_{i}$ denote the OOP healthcare payment of the ${i}^{th}$ patient and $I$ the annual income of the patient. A catastrophic payment is considered to have occurred if the share of yearly income spent on OOP expenses surpasses the catastrophic threshold, expressed as, $1(\frac{P}{I}> {Z}_{cat} )$. Determining the appropriate catastrophic threshold empirically presents a challenge and often depends on the chosen denominator for assessing financial catastrophe. Varying thresholds have been proposed in some cases [26]. The incidence of financial catastrophe or the catastrophic payment headcount is defined as follows:

1 $$H= \frac{1}{n} \sum_{i=1}^{n}{E}_{i} = {\mu }_{E}$$

where n is the total number of households, ${E}_{i}$ represents the out-of-pocket healthcare payment for the ${i}^{th}$ household, and ${\mu }_{E}$ is the mean of ${E}_{i}$. This measure, however, does not capture the extent to which the share of household income spent on OOP expenses exceeds the threshold. To address this, a catastrophic payment gap is computed. This gap measure quantifies the average amount by which OOP healthcare payments exceed the specified catastrophic threshold, providing a more detailed understanding of the financial burden on households.

### Catastrophic payment gap index

This measure captures the average extent to which OOP healthcare payments as a proportion of income exceed the threshold ${Z}_{cat}$. Formally, if we define ${O}_{i}=\frac{{P}_{i}}{{I}_{i}}- {Z}_{cat}$ as the extent of overshoot, which is zero or for those that do not incur catastrophic payments, then the catastrophic payment gap may be specified as

2 $$G=\frac1n\sum\limits_{i=1}^nO_i=\mu_{O\;}for\;O_i>0$$

where ${\mu }_{O}$ is the mean of ${O}_{i}$.

Since the catastrophic payment gap is averaged across all households, regardless of whether they experience financial catastrophe, it does not fully represent the severity of the payments among those who do incur them. To address this, the mean positive gap (MPG) is calculated to reflect the average catastrophic gap specifically among households that experience financial catastrophe. This essentially provides a more accurate measure of the financial burden for those households used to compute the headcount in the initial equation [25, 26].

3 $$MPG= \frac{\sum_{i=1}^{n}{o}_{i} }{\sum_{i=1}^{n}{E}_{i} }= \frac{{\mu }_{o}}{{\mu }_{E}}$$

The previously mentioned approaches do not distinguish between poor and affluent households in terms of exceeding the threshold [24, 25]. However, it can be argued that most societies, including Ghana, are more concerned when households in the lowest income quintile face financial catastrophe compared to wealthier households. Although there are different methods to address this concern, this paper proposes a weighted version of all indices (headcount and gap) that accounts for whether it is primarily the poorer or wealthier households that surpass the threshold. This method provides one solution, but an alternative approach could involve using variable thresholds, assigning lower thresholds to poorer households relative to wealthier ones [17, 26].

### Weighted catastrophic payment headcount and weighted catastrophic gap indices

To obtain these indices, the concentration indices of ${E}_{i}$ and ${O}_{i}$ are computed such that ${C}_{E}$ is the concentration index of ${E}_{i}$ and ${C}_{o}$ is the concentration index of ${O}_{i}.$ A negative concentration index ${(C}_{E}<0 or {C}_{o}<0)$ means that such payments are concentrated among poorer households. Weighted catastrophic payment headcount is obtained as:

4 $$W_{cat}^E=\mu_{E\;}\left(1-C_E\right)$$

If ${W}_{cat }^{E}>{H}_{cat}$ it means that poorer households exceed the threshold more but if ${W}_{cat }^{E}{<H}_{cat}$ then the incidence of catastrophic spending is concentrated on the rich [15, 27]. If poorer households tend to exceed the threshold more, the concentration index C_E_ will be negative, and this will raise ${W}_{Cat}^{E}$ above ${H}_{Cat}$. The reverse is the case when C_E_ is positive [25]. Weighted catastrophic payment gap on the other hand is obtained as

5 $$W_{cat}^G=\mu_{o\;}\left(1-C_o\right)$$

If ${W}_{cat }^{G}>{G}_{cat}$ then large excesses tend to be concentrated among the poor, but the reverse is the case when ${W}_{cat }^{E}<{H}_{cat}$ [26].

To compute these weighted indices, the method used is based on the approach proposed by Wagstaff and van Doorslaer [25]. The concentration index provides a summary measure of the inequality in catastrophic payments, weighted by income distribution, allowing for the adjustment of these indices to reflect the disproportionate impact on poorer or wealthier households. This approach ensures that the financial burden is captured in a more nuanced manner by accounting for the relative income distribution of households incurring catastrophic expenditures.

### Regression analysis

To examine the factors associated with the likelihood of experiencing catastrophic health expenditures (CHE), we used logistic regression models. The dependent variable in the analysis was a binary indicator of whether a patient experienced CHE, defined as healthcare expenses exceeding 10%, 20%, 30%, and 40% of annual income. This approach follows similar studies that have employed logistic regression to investigate the determinants of CHE in various settings [28, 29].

The model is specification as follows:

6 $$\begin{aligned}logit\left(P\left({CHE}_{i}=1\right)\right)&=In\left(\frac{P\left({CHE}_{i}=1\right)}{1-P\left({CHE}_{i}=1\right)}\right)\\&={ \beta }_{0}+ { \beta }_{1}{Age}_{i}+ { \beta }_{2}{Sex}_{i}+{ \beta }_{3}{Education}_{i }+ { \beta }_{3}{Severity}_{i }\\&+ { \beta }_{4}{FractureType}_{i }+ { \beta }_{5}{NHIS}_{i}\end{aligned}$$

The independent variables in the model include age (categorized into < 40, 41–60, and 60 + years), sex (male/female), and educational level (no education, primary, secondary, post-secondary). Additionally, the model incorporates the severity of injury (moderate/severe, based on the Injury Severity Score (ISS)), type of fracture (humerus, radius and ulna, femur, tibia), and National Health Insurance Scheme (NHIS) coverage (yes/no). By including demographic and clinical characteristics in the model, this analysis aims to uncover the key drivers of financial hardship among patients with long bone fractures in Ghana.

## Results

### Demographic and clinical characteristics

Table 1 reports data from 2,980 patients with long bone fractures, who responded to questions about their annual income and OOP expenses. Among these patients, 72.58% (*n* = 2,163) were male, and 27.42% (*n* = 817) were female. The age distribution showed that the majority of patients (65.54%, *n* = 1,031) were between 14–40 years, followed by 27.21% (*n* = 428) aged 41–60 years, and 7.25% (*n* = 114) who were 60 years or older.

**Table 1:** Descriptive statistics of patients

Variable	Frequency	Percentage	Average OPP($)
**Sex**			
Female	817	27.42	271.63
Male	2163	72.58	343.68
**Age**			
14–40 years	1031	65.54	289.73
41–60 years	428	27.21	346.08
60 years +	114	7.25	281.56
**Hospital**			
Cape Coast Teaching Hospital	1070	35.88	394.51
Komfo Anokye Teaching Hospital	40	1.34	52.26
Korle Bu Teaching Hospital	946	31.72	399.93
Tamale Teaching Hospital	924	31.05	125.68
**Educational Level**			
No education	308	19.43	136.82
Primary education	604	38.11	297.25
Secondary education	380	23.97	282.55
Post-Secondary education	293	18.49	203.48
**Bone fractured**			
Humerus	2569	86.85%	284.87
Radius and Ulna	331	11.19%	570.92
Femur	48	1.62%	458.97
Tibia	10	0.34%	1034.10
**Type of Bone Fracture**			
Open	380	27.78	349.66
Closed	988	72.22	242.65
**Severity Score**			
Not Severe	1892	86.95	270.8
Severe	284	13.05	404.14
**Region**			
North	2071	69.59	398.27
South	905	30.41	125.01
**Type of settlements**			
Urban	1566	80.68	297.06
Rural	375	19.32	220.33
**NHIS Coverage**			
Yes	2221	74.53	307.64
No	759	25.47	384.84

The total number of patients used in the analysis is 2,980. For variables with fewer observations, it means some patients did not answer those specific questions. Average OOP payments were calculated based on the exchange rate of GHS 5.08 per U.S. dollar for the period July 2017 to July 2020

In terms of hospital distribution, the largest proportion of patients received treatment at Cape Coast Teaching Hospital (35.88%, *n* = 1,070), followed by Korle Bu Teaching Hospital (31.72%, *n* = 946), and Tamale Teaching Hospital (31.05%, *n* = 924). Komfo Anokye Teaching Hospital treated the fewest patients, accounting for just 1.34% (*n* = 40) of the total sample.

Regarding educational attainment, 38.11% (*n* = 604) of patients had completed primary education, while 23.97% (*n* = 380) had secondary education, and 18.49% (*n* = 293) had post-secondary education. A smaller percentage, 19.43% (*n* = 308), had no formal education. The most common type of fracture involved the humerus (74.79%, *n* = 2,569), followed by fractures of the radius and ulna (11.27%, *n* = 331), femur (3.22%, *n* = 48), and tibia (0.54%, *n* = 10).

The majority of fractures were classified as not severe (86.95%, *n* = 1,892), while 13.05% (*n* = 284) were categorized as severe. Geographically, most patients were from northern Ghana (69.59%, *n* = 2,071), with the remaining 30.41% (*n* = 905) residing in southern regions. Additionally, the majority of patients lived in urban areas (55.70%, *n* = 1,566), while rural and peri-urban residents accounted for 19.48% (*n* = 375) and 24.82% (*n* = 483), respectively.

Finally, 74.53% (*n* = 2,221) of the patients were covered by the National Health Insurance Scheme (NHIS), while 25.47% (*n* = 759) did not have insurance coverage.

### Average out-of-pocket payments by demographic characteristics

Table 1 provides an overview of the average out-of-pocket payments (OOP) across various demographic and clinical characteristics. The analysis shows notable variation in OOP payments by gender, age, hospital, education, fracture type, and NHIS coverage status.

On average, male patients had higher OOP payments ($343.68) compared to female patients ($271.63). In terms of age, patients between 41–60 years had the highest average OOP payments ($346.08), followed by those aged 14–40 years ($289.73) and those aged 60 years and above ($281.56).

Hospital-specific differences were also observed, with patients at Korle Bu Teaching Hospital incurring the highest average OOP payments ($399.93), followed closely by patients at Cape Coast Teaching Hospital ($394.51). Tamale Teaching Hospital had notably lower OOP payments ($125.68), and the lowest payments were recorded at Komfo Anokye Teaching Hospital ($52.26).

Education level appeared to influence OOP payments, with patients with primary education paying the highest average OOP ($297.25), followed by those with secondary education ($282.55) and post-secondary education ($203.48). Interestingly, patients with no education incurred the lowest OOP payments, averaging $136.82.

When examining fracture types, patients with tibia fractures had the highest average OOP payments ($1,034.10), significantly higher than those with radius and ulna fractures ($570.92), femur fractures ($458.97), and humerus fractures ($284.87).

Finally, NHIS coverage reduced the financial burden on patients, with those covered by NHIS incurring lower average OOP payments ($307.64) compared to uninsured patients, who faced higher payments ($384.84).

### Catastrophic healthcare expenditure

CHE were assessed using yearly income thresholds of 10%, 20%, 30%, and 40%. The incidence of CHE was highest at the 10% threshold (53.21%) and decreased as the threshold increased to 20% (32.12%), 30% (22.80%), and 40% (16.63%). The negative concentration indices, ranging from −0.17 to −0.239, indicated that CHE disproportionately affected lower-income patients (Table 2).

**Table 2:** Catastrophic healthcare expenditure

	**Catastrophic healthcare expenditure on bone fractures using yearly income**			
**Thresholds**	**10%**	**20%**	**30%**	**40%**
**Headcount**				
Catastrophic expenditure headcount	53.21%	32.12%	22.80%	16.63%
Concentration index	−0.17	−0.219	−0.223	−0.239
Weighted headcount	62.42%	39.19%	27.89%	20.62%
**Gap measures**				
Catastrophic payment headcount	30.15%	37.17%	40.68%	44.15%
Concentration index	−0.161	−0.164	−0.212	−0.260
Weighted headcount	35.01%	43.27%	49.30%	55.52%

A total of 2,980 patients were used in this analysis

The weighted headcount measures, which account for income distribution, showed a higher prevalence of CHE among poorer households, with rates of 62.42% at 10%, 39.19% at 20%, 27.89% at 30%, and 20.62% at 40%. The catastrophic payment gap increased with higher thresholds, indicating growing financial distress among patients. The weighted gap measures further highlighted that poorer patient experienced more significant financial burdens, with weighted headcount values of 35.01% at 10%, 43.27% at 20%, 49.30% at 30%, and 55.52% at 40%.

### Risk factors for CHE

The logistic regression analysis (Table 3) reveals key factors influencing the likelihood of experiencing CHE across different income thresholds (10%, 20%, 30%, and 40%). At all thresholds, being male was not significantly associated with the likelihood of experiencing CHE compared to the base category (female). The coefficients for males were close to zero, with none reaching statistical significance, indicating no substantial gender-based differences in CHE risk.

**Table 3:** Risk Factors for CHE at different income thresholds

	**CHE Headcount Thresholds**			
**Variables**	**10%**	**20%**	**30%**	**40%**
**Gender**				
Male	−0.0108	0.0217	0.00250	0.000988
	(0.0437)	(0.0390)	(0.0342)	(0.0285)
Age				
41–60 years	−0.107**	−0.0307	−0.0109	−0.0232
	(0.0416)	(0.0370)	(0.0329)	(0.0252)
60 years +	−0.156**	−0.0443	−0.00734	0.0276
	(0.0681)	(0.0586)	(0.0550)	(0.0524)
Education				
Primary	−0.132**	−0.0946*	−0.0372	−0.0190
	(0.0521)	(0.0530)	(0.0497)	(0.0452)
Secondary	−0.196***	−0.162***	−0.104**	−0.0863**
	(0.0580)	(0.0560)	(0.0476)	(0.0403)
Post-Secondary	−0.483***	−0.249***	−0.117**	−0.0738*
	(0.0510)	(0.0521)	(0.0499)	(0.0437)
Region				
North	−0.162***	−0.109***	−0.0883**	−0.0648**
	(0.0411)	(0.0394)	(0.0358)	(0.0310)
Type of bone Fracture				
Humerus	-	−0.431*	−0.535**	−0.387*
		(0.230)	(0.213)	(0.210)
Radius and Ulna	-	−0.373	−0.460**	−0.335
		(0.232)	(0.215)	(0.210)
Femur	-	−0.189	−0.349	−0.275
		(0.252)	(0.233)	(0.218)
**Severity Score**				
Severe	0.251***	1.204***	0.188***	0.133***
	(0.0487)	(0.0534)	(0.0514)	(0.0437)
Open	0.105***	0.110***	0.0799**	0.0428*
	(0.0399)	(0.0371)	(0.0333)	(0.0260)
NHIS				
Yes	0.171**	0.0592	0.0367	0.0152
	(0.0693)	(0.0556)	(0.0464)	(0.0380)
Location				
Rural	−0.0808*	−0.0369	0.0327	0.0556*
	(0.0474)	(0.0365)	(0.0349)	(0.0310)
Observations	658	663	663	663

Robust standard errors are in parentheses. *** *p* < 0.01, ** *p* < 0.05, * *p* < 0.1. Coefficients represent the average marginal effects after estimating the logit model. The discrete change from the base level is shown for factor levels. Base categories: Age (< 40 years), Gender (Female), Education (No education), Region (South), Severity Score (Not Severe), Type of Fracture (Tibia), Fracture Type (Closed), NHIS (No), and Location (Urban)

At all thresholds, being male was not significantly associated with the likelihood of experiencing Catastrophic Health Expenditure (CHE) compared to the base category (female). The coefficients for males were close to zero across all thresholds, with none reaching statistical significance. This indicates that there was no substantial gender-based differences in the risk of CHE, suggesting that both males and females faced similar financial burdens related to long bone fractures.

In contrast, age emerged as a significant predictor of CHE, particularly for patients aged 41–60 years and those over 60 years. At the 10% threshold, patients aged 41–60 were significantly less likely to experience CHE compared to those under 40 years (the base category), with a marginal effect of −0.107 (*p* < 0.05). A similar trend was observed for patients aged over 60 years at the 10% threshold, with a marginal effect of −0.156 (*p* < 0.05). However, the significance of age diminished at higher thresholds, suggesting that older age groups are more likely to face financial distress at lower income levels but show no significant difference from younger patients at higher thresholds.

Educational level also played a critical role in reducing the likelihood of CHE across all thresholds. Patients with primary education were significantly less likely to experience CHE at the 10% threshold (marginal effect = −0.132, *p* < 0.05) and the 20% threshold (marginal effect = −0.0946, *p* < 0.1) compared to those with no education. Moreover, this protective effect of education became even stronger for those with secondary and post-secondary education. For example, patients with secondary education had a marginal effect of −0.196 (*p* < 0.01) at the 10% threshold, while those with post-secondary education showed a marginal effect of −0.483 (*p* < 0.01). These findings underscore the importance of educational attainment in mitigating the risk of financial distress due to healthcare expenditures.

Regional differences also significantly influenced the risk of CHE. Patients living in the northern regions of Ghana were significantly less likely to experience CHE compared to those living in the south (the base category). This effect was most pronounced at the 10% threshold, with a marginal effect of −0.162 (*p* < 0.01), and remained significant at higher thresholds (e.g., −0.0648 at the 40% threshold, *p* < 0.05). These results suggest that patients in the northern region are less financially vulnerable to healthcare costs compared to those in the southern regions.

The type of bone fracture had a notable impact on CHE risk. For patients with humerus and radius and ulna fractures, there was a significant reduction in the likelihood of CHE compared to the base category (tibia fractures), particularly at higher thresholds. At the 30% threshold, patients with humerus fractures had a marginal effect of −0.535 (*p* < 0.05), and those with radius and ulna fractures had a marginal effect of −0.460 (*p* < 0.05), indicating a lower risk of financial distress. Injury severity was one of the strongest predictors of CHE. Patients with severe injuries were significantly more likely to incur CHE across all thresholds, with a marginal effect of 0.251 (*p* < 0.01) at the 10% threshold and 0.133 (*p* < 0.01) at the 40% threshold. Additionally, patients with open fractures were more likely to experience CHE compared to those with closed fractures (the base category). For instance, at the 10% threshold, open fractures had a marginal effect of 0.105 (*p* < 0.01), indicating the significant financial burden associated with these more complex injuries.

Finally, NHIS coverage was found to be a significant protective factor against CHE, but only at the lower thresholds. At the 10% threshold, patients with NHIS coverage were less likely to experience CHE, with a marginal effect of 0.171 (*p* < 0.05). However, this protective effect diminished at higher thresholds, suggesting that NHIS may provide more effective financial relief for lower-income patients but becomes less effective as the severity of financial burden increases.

In terms of location, living in rural areas was associated with a lower risk of CHE at the 10% threshold, with a marginal effect of −0.0808 (*p* < 0.1). However, this trend reversed at higher thresholds, where rural residents were more likely to experience CHE (marginal effect = 0.0556, *p* < 0.1 at the 40% threshold).

## Discussion

The results of this study highlight the substantial financial burden experienced by patients with long bone fractures, particularly through OOP payments and CHE. Among the 2,980 patients included in the analysis, the majority were male (72.58%) and aged between 14–40 years (65.54%). Average OOP payments varied significantly across demographic and clinical categories, with male patients incurring higher OOP payments than females. Additionally, patients with tibia fractures faced the highest financial burden, averaging $1,034.10 in OOP payments, whereas patients with humerus fractures had much lower average costs. Geographically, those treated at Cape Coast and Korle Bu Teaching Hospitals had significantly higher OOP payments compared to patients at Komfo Anokye and Tamale Teaching Hospitals.

The incidence of CHE in this study was notably high, particularly at the 10% income threshold, where 53.21% of patients experienced catastrophic expenditures. As the income threshold increased, the incidence of CHE declined, but even at the 40% threshold, 16.63% of patients still faced CHE. Lower-income households were disproportionately affected, as indicated by negative concentration indices across all thresholds. These findings underscore the significant financial vulnerability faced by patients with long bone fractures and the pressing need for interventions to alleviate this burden.

The high incidence of CHE in this study, with over 53% of patients affected at the 10% threshold, reflects a broader global trend of financial vulnerability in trauma care, as seen in both LMICs and high-income countries. In LMICs, studies like those by Bolongaita et al. [30] and Njagi et al. [31] emphasize that OOP payments disproportionately burden poorer households, mirroring our findings. Twea et al. [32] similarly found significant orthopedic costs in Malawi, further supporting the idea that trauma care imposes an unsustainable financial burden in resource-limited settings. In high-income countries, studies by Bonafede et al. [33] and Billig et al. [34] show that uninsured and vulnerable populations also face high risks of CHE, indicating that financial protection gaps persist globally. The consistency of these findings across income contexts underscores the urgent need for targeted interventions such as expanding insurance coverage and reducing OOP payment to alleviate the financial burden of trauma care worldwide. The findings on CHE in patients with long bone fractures reveal critical policy implications, particularly the inadequacy of current financial protection mechanisms like the NHIS, which offers limited relief from OOP costs. This aligns with other studies, such as Vanderkarr et al. [35], which highlight that insurance often provides only partial relief for non-union cases of long bone fractures. Similarly, Mulaga et al. [36] in Malawi found that despite free public healthcare, 13.7% of households incurred CHE, reflecting the persistent financial burden of OOP payments. Khan et al. [37] in Bangladesh further demonstrated that OOP spending increased poverty by 3.5%, underscoring the insufficiency of existing insurance schemes. These studies mirror our findings, where NHIS in Ghana, though helpful, remains inadequate, particularly for severe cases requiring surgery. These comparisons highlight the urgent need for policy reforms to expand coverage and reduce OOP costs, ensuring financial protection for vulnerable populations in both low- and middle-income settings.

Expanding the coverage scope of NHIS or introducing targeted financial assistance, such as subsidies for high-cost procedures related to severe injuries, could significantly reduce the financial burden on vulnerable populations [38, 39]. This aligns with global efforts advocating universal health coverage to protect individuals from financial hardship due to medical expenses. Studies have shown that uninsured patients, in particular, face higher risks of CHE, emphasizing the need for more inclusive health policies [40, 41]. Addressing these gaps through policy reform could mitigate the financial vulnerability of patients, especially those in low-resource settings.

The results of this study show a clear gender disparity in OOP payments for long bone fractures, with male patients incurring significantly higher costs ($343.68) compared to females ($271.63). This discrepancy likely stems from the fact that males, particularly those engaged in physically demanding and high-risk occupations, tend to experience more severe injuries, which require costlier medical interventions. Ali et al. [42] similarly found that men often incur higher healthcare costs for osteoporotic fractures due to the severity of their injuries and their healthcare-seeking behavior. Onizuka et al. [43] and Li et al. [44] further support this by highlighting that men, especially in high-risk jobs, are prone to more severe injuries and are less likely to seek early treatment for conditions like osteoporosis, exacerbating their medical costs.

These findings are echoed in high-income settings as well, where studies such as Nishtala et al. [45] and Boz et al. [46] demonstrate that male patients, particularly uninsured and lower-income individuals, face disproportionately higher financial burdens due to the nature of their injuries. This parallel suggests that regardless of the setting—whether in LMICs like Ghana or high-income countries—the intersection of gender, occupation, and injury severity plays a significant role in healthcare costs. These results highlight the importance of gender-targeted interventions, such as improved occupational safety measures and early diagnosis programs for men, to mitigate the financial burden of fractures and address these disparities in healthcare costs.

In terms of socioeconomic disparities, patients with higher educational attainment experienced lower rates of CHE, suggesting that better education may provide individuals with improved access to resources and preventive care. This finding aligns with multiple studies that emphasize the protective effect of education on financial health outcomes. For instance, Mohsin et al. [47] found that higher educational attainment is associated with lower rates of CHE, as individuals are more likely to access preventive healthcare services and manage their healthcare costs more effectively. Similarly, Valentin et al. [48] reported that individuals with higher socioeconomic status, including better education, are less likely to experience fragility fractures and associated financial burdens. Additionally, Morenz et al. highlighted that lower educational levels contribute to increased healthcare spending and greater financial barriers, further supporting the link between education and reduced financial distress [49]. Together, these studies underscore the role of education in mitigating the financial impact of healthcare expenses, particularly for patients with long bone fractures.

Geographically, patients from northern regions in this study had lower OOP payments compared to their southern counterparts. This disparity may be attributed to differences in healthcare access and cost structures, which have been observed in other regions as well. For instance, Cyr et al. [50] highlighted that rural and less-resourced areas tend to face greater barriers to accessing specialty care, which can affect healthcare costs and treatment options. Similarly, Wallace et al. [51] reported that regional and demographic factors, such as race and socioeconomic status, significantly influence healthcare access and associated costs, with more pronounced disparities in under-resourced regions. Wray et al. [52] further supported this by showing that geographic location, along with insurance type, can lead to substantial differences in healthcare satisfaction and costs. These studies provide a broader context for understanding the geographical disparities observed in this study and emphasize the need for targeted interventions to reduce the financial burden on patients in less accessible regions.

### Limitations of the study

One significant limitation of this study is the disclosure of financial status by patients. In Ghana, as in many other settings, patients often underreport their actual earnings, while those with very low incomes may occasionally report higher figures to feel better about their financial situation. This discrepancy in income reporting could affect the accuracy and generalizability of our findings. Underreporting income may lead to an overestimation of the incidence of CHE, while overreporting by lower-income individuals could underestimate the true financial burden they face. Also, the study did not account for the cost of surgical implants in the OOPP calculations, which could further skew the assessment of the financial impact on households.

Additionally, the cross-sectional design of the study limits our ability to establish causal relationships between the identified factors and CHE. Despite these limitations, the findings provide valuable insights into the financial burdens associated with long bone fractures and can serve as a significant guide for policymakers and surgeons. By addressing these limitations in future research, more accurate and comprehensive data can be obtained, further informing policy interventions and healthcare practices.

### Recommendations for future research

Future research should aim to address the limitations identified in this study (especially cost of implants which are usually high) and further explore the financial burden of long bone fractures and other orthopaedic conditions. Longitudinal studies are needed to track the financial impact of long bone fractures over time, providing a better understanding of long-term economic consequences. Improved methods for collecting accurate income data, such as verifying self-reported income through multiple sources, are essential to enhance the accuracy of findings.

Investigating cost-effective treatment methods for long bone fractures is crucial to identifying approaches that reduce financial burdens without compromising patient care. Examining the impact of different types and levels of insurance coverage on the financial burden of orthopaedic conditions can assess the adequacy of current coverage options. Research should also explore socioeconomic disparities in financial burden, focusing on how income, education, and geographic location influence the likelihood of experiencing catastrophic health expenditures.

Evaluating the effectiveness of patient support programs, such as financial counselling and assistance programs, can provide insights into additional support mechanisms for patients facing significant financial challenges. Expanding research to include a broader range of orthopaedic conditions, such as joint replacements and spinal surgeries, can offer a more comprehensive understanding of the economic challenges faced by orthopaedic patients. By addressing these areas, future research can contribute to more effective policy interventions and healthcare practices, improving financial protection and overall well-being for patients with orthopaedic conditions.

## Conclusion

In conclusion, this study highlights the significant financial burden of long bone fractures on patients in Ghana, with a high incidence of CHE observed, particularly at lower thresholds. The findings indicate that despite some relief provided by the NHIS, the coverage remains insufficient to offer substantial financial protection, especially for severe cases requiring surgical intervention. Gender and educational disparities were evident, with male patients and those with lower educational attainment facing higher OOP payments and greater risk of CHE. Regional differences also emerged, with patients in the northern regions experiencing lower OOP payments compared to those in the south, pointing to geographic inequities in healthcare access and cost structures.

The study underscores the urgent need for policy interventions, such as expanding NHIS coverage, introducing targeted financial assistance for high-cost treatments, and addressing regional disparities in healthcare provision. Future research should explore the long-term financial impact of fractures, improve income data accuracy, and investigate cost-effective treatment methods. These steps can help reduce the financial burden on vulnerable populations and enhance overall financial protection for patients with long bone fractures, contributing to more equitable healthcare outcomes in Ghana.

## Data Availability

The datasets used and/or analysed during the current study are available from the corresponding author on reasonable request.

## Appendix

**Table 4:** Descriptive statistics

Variable	Obs	Mean	Std. Dev.	Min	Max
Annual Income	2980	8564	11721.889	600	144000
OPP	2980	1398	3246.592	0	198080

Source: Authors’ own construction based on survey data
